# 

**Published:** 1895-12

**Authors:** 


### CONTENTS.

ORIGINAL COMMUNICATIONS—

SURGICAL INTERFERENCE IN RECTAL DISORDERS. By J. McFadden Gaston, M.D., Atlanta, Ga. ... 577

INTRA-CAPSULAR FRACTURES OF THE NECK OF THE FEMUR. By G. A. Baxter, A.M., M.D., Chattanooga, Tenn. ... 583

REMARKS ON GOITRE, WITH REPORT OF A CASE. By Joseph A. White, A.M., M.D., Richmond, Va. ... 593

THE LESIONS ASSOCIATED WITH DYSMENORRHEA. By William S. Gardner, M.D., Baltimore, Md. ... 599

SOCIETY REPORTS—

SEVENTH ANNUAL MEETING OF THE TRI-STATE MEDICAL SOCIETY OF ALABAMA, GEORGIA AND TENNESSEE ... 602

CORRESPONDENCE—

MALARIAL HEMATURIA ... 608

TEN RULES GOVERNING THE ADMINISTRATION OF ETHER ... 610

DEPARTMENT AND SECRETARY OF PUBLIC HEALTH ... 611

EDITORIAL—

DR. ROBERT BATTEY ... 613

THE SOUTHERN MEDICAL AND GYNECOLOGICAL ASSOCIATION ... 615

SELECTIONS AND ABSTRACTS—

GENERAL MEDICINE AND THERAPEUTICS ... 618

SKIN DISEASES AND SYPHILIS ... 625

SURGERY ... 627

MEDICAL ITEMS ... 635

BOOK REVIEWS ... 638

ADVERTISERS' INDEX TO ADVERTISEMENTS ... xiv

MEDICAL ITEMS—CONTINUED ... x, xi, xii, xiii

CLASSIFIED INDEX TO ADVERTISEMENTS ... xxxiii

